# Pericarditis recurrence is associated with milder electrocardiographic, echocardiographic, and laboratory findings

**DOI:** 10.1007/s11739-024-03579-7

**Published:** 2024-03-29

**Authors:** Tal Weiss, Edward Itelman, Dor Lotan, Amitai Segev, Dov Freimark, Michael Arad, Yishay Wasserstrum

**Affiliations:** 1 Leviev Heart Center, Sheba Medical Center, 2 Derech Sheba St, Tel-Ha’shomer, 52662 Ramat Gan, Israel; 2 Faculty of Medicine, Tel-Aviv University, Tel Aviv, Israel; 3 Cardiology Division, Rabin Medical Center, Petach-Tikva, Israel; 4 Columbia University, New-York City, NY USA

**Keywords:** Pericarditis, Acute pericarditis, Recurrent pericarditis

## Abstract

Recurrent pericarditis (RP) complicates approximately 30% of acute pericarditis (AP) cases. We sought to compare the prevalence and severity of objective findings seen in patients with RP. A retrospective single-center study during 2010–2019, including 765 patients diagnosed with AP. Clinical, electrocardiographic, echocardiographic, and laboratory findings were extracted from the local electronic health records. Recurrence during follow-up was documented in 134 patients (17.5%), with a median time to recurrence of 101 (± 59–251) days. The median age was 60 years (IQR 45–72), 68% were male. Most patients were defined as having idiopathic\viral pericarditis (64%). The clinical manifestation during the recurrent event of pericarditis was less prominent or attenuated when compared to the initial event—ECG signs (ST elevation 12% vs. 26%; *p* = 0.006, Knuckle sign 13% vs. 33%; *p* < 0.001, ST larger in lead L2 than L3 4% vs. 19%; *p* < 0.001), pericardial effusion moderate and above (11% vs. 30%; *p* = 0.02), and inflammatory markers (mean peak CRP levels 66 mg/l vs. 97 mg/l; *p* < 0.001). Similar results were seen in the subgroup of patients defined as having idiopathic\viral pericarditis. Up to 20% of patients who did not have ECG signs or a significant pericardial effusion in their 1st event demonstrated these findings during the recurrence, though still to a lesser extent compared with those who had these signs in their 1st event. The objective findings of AP are less pronounced during recurrent events. Future studies should focus on the role of advanced biomarkers and imaging in defining true RP events.

## Introduction

**Fig. 1:**
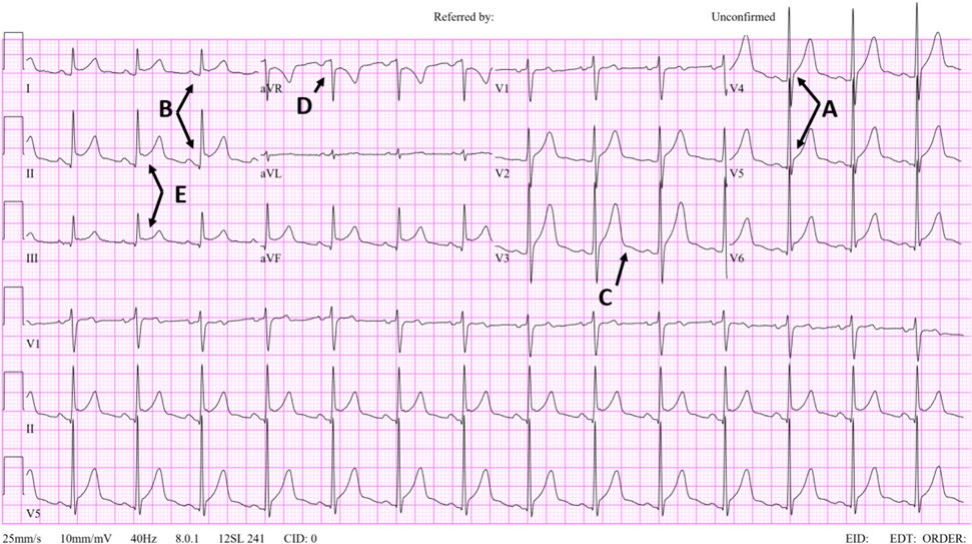
An electrocardiogram showing findings typical of acute pericarditis. **A**: ST elevations; **B**: PR depressions; **C**: Spodick’s sign; **D**: Knuckle sign; **E**: ST elevations in L2 greater than L3

Acute pericarditis (AP) is an inflammatory disease of the pericardium. The reported prevalence of pericarditis in the United States is 28 cases per 100,000 subjects per year, and it constitutes approximately 5% of all referrals to Emergency departments for chest pain and 0.2% of all cardiovascular in-hospital admissions [1]. The 2015 ESC Guidelines for the diagnosis and management of pericardial diseases recommend a diagnostic rule based on the presence of 2 of: typical chest pain, pericardial friction rub, typical ECG changes, and evidence of a pericardial effusion. The classical evolution of the ECG has 4 stages: stage 1 (PR depression and ST-segment elevation), stage 2 (ST-segment normalization), stage 3 (T-wave inversion), and stage 4 (ST-T normalization). However, this typical evolution has been previously reported in no more than 60% of cases [2]. Spodick’s sign, a downsloping morphology of the T–P interval, has been described as present in 80% of patients with pericarditis [3]. Along with Spodick’s sign, several electrocardiographic findings have been reported as useful in diagnosing pericarditis including PR segment depression, an ST elevation in lead II greater than lead III, and the knuckle sign, defined as a PR elevation and ST depression on lead aVR [4, 5] (Fig. 1).

Recurrent pericarditis (RP) is the most common complication following the initial episode of pericarditis [6] and is likely the most troublesome. It is defined as the recurrence of symptoms and signs of pericarditis after a symptom-free interval of 4–6 weeks [2]. RP prevalence has been described as 15–30%, and a third event occurs in 20–40% of cases. Up to 10% of patients suffer from multiple recurrences and may require long-term or permanent corticosteroid therapy [2, 7]. Novel therapeutic drugs, such as interleukin-1 (IL-1) antagonists, have been introduced for the management of these patients [8–13].

Cardiac imaging is an integral part of the diagnostic and staging process of pericarditis. Echocardiography is the most commonly used imaging test in the diagnosis of AP. New modalities such as cardiac magnetic resonance imaging (CMR) can be used when echocardiographic findings are ambiguous or in case of myocardial involvement [14–16]. An observational study on 275 patients showed that about 10% of patients with a previous diagnosis of AP may complain of recurrent pain during the follow-up, without evidence of pericardial inflammation according to traditional criteria. However, while the same patients had a higher risk of recurrence over a 40-month follow-up [17], data comparing the objective findings of AP in recurrent events is lacking.

We sought to describe the variation in the severity of clinical and laboratory features between the first presentation of AP and the first documented recurrent event, in a contemporary cohort of patients with AP either secondary to an idiopathic\viral etiology or post-pericardiotomy syndrome (PPS).

## Methods

This was a single-center retrospective study performed in a tertiary medical center in Israel, reviewing admissions during 2010–2019. The study included patients ≥ 18 years old admitted with the diagnosis of acute pericarditis. From this population, we selected those who had both documented acute and recurrent events to compare their clinical features during the two events. A recurrence was defined as the recurrence of symptoms and signs of pericarditis after a symptom-free interval of 4–6 weeks following an initial episode of pericarditis. Exclusion criteria consisted of a previous history of acute pericarditis, asymptomatic pericardial effusion, chronic, persistent, or constrictive pericarditis, and active malignancy. We also excluded patients in which the main manifestation was of myocardial inflammation (i.e., perimyocarditis), and those with elevated troponin I as defined by our biochemical laboratory standards, based on the available measurement kits at the time, mostly defined as under 59 ng/ml. The exception to this rule was a handful of PPS cases presenting early in the post-operative phase, where the troponin levels were disregarded. Patients eventually determined to have anginal chest pain after further invasive or non-invasive investigations were also excluded.

The statistical analysis was carried out with the use of R version 4.2.3 software (The R Foundation) and R-studio 2023.03.0 (RStudio, Inc). Categorical variables were reported in frequencies and percentages, and the difference between paired groups were tested with the Mc-Nemar test. Continuous variables were explored using a histogram plot and the Shapiro–Wilk test. Variables found to have a normal distribution were reported as mean and standard deviation values, and the difference between groups was tested with the paired T-test method when appropriate. Continuous variables that did not have a normal distribution were reported as median and interquartile range (IQR, 25th–75th percentiles), and the difference between groups was tested with the Wilcoxon rank test for related samples, respectively, when appropriate.

## Results

In our final analysis, we included 765 patients with a documented event of pericarditis that met the inclusion criteria. The median age was 60 years (IQR 45–72), with a male predominance (68%). Most patients were defined as having idiopathic\viral pericarditis (64%, 486 patients). Other etiologies included 28% post-pericardiotomy\post-myocardial injury, 5% secondary to connective tissue disease, 2% uremic, and 0.5% familial Mediterranean fever. Nine patients (1.2%) had pericarditis in the setting of a complex clinical scenario, making the etiology of these cases indeterminate or unclear. Recurrence during follow-up was documented in 134 patients (17.5%), with a median time to recurrence of 101 (± 59–251) days.

The prevalence of various ECG findings associated with AP was higher at the 1st presentation (Table 1). There was a significantly higher rate of ST elevation during the first event compared to subsequent recurrence (26% in the first event vs. 12% in the recurrence; *p* = 0.006). Similarly, for the presence of Knuckle sign (33% in the first event vs. 13% in the recurrence; *p* < 0.001) and the presence of ST elevation larger in lead L2 than L3 (19% in the first event vs. 4% in the recurrence; *p* < 0.001).

**Table 1:** Comparison of the clinical parameters during acute pericarditis and the recurrent event: entire cohort

	First event	Recurrence	*p*- value
Mean initial CRP levels (mg/l)	69 ± 54	51 ± 40	0.03
Mean maximal CRP levels (mg/l)	97 ± 75	66 ± 52	< 0.001
ST elevations *n* (%)	34 (26%)	16 (12%)	0.006
PR depressions *n* (%)	58 (45%)	46 (34%)	0.08
Spodick’s sign *n* (%)	23 (18%)	16 (12%)	0.27
Knuckle sign *n* (%)	43 (33%)	18 (13%)	< 0.001
ST elevation L2 > L3 *n* (%)	25 (19%)	6 (4%)	< 0.001
Pericardial effusion > small *n* (%)	34 (30%)	6 (11%)	0.02

CRP, C-reactive protein

Our patients were mostly treated according to current guidelines. During the 1st event, NSAIDs were given in 48% of cases, steroids were given in 58% of cases, and colchicine was given in 80% of cases. Pharmacologic management during the 1st event was mostly not associated with the presence of ECG findings or a significant pericardial effusion during the recurrent event.

When looking solely at the idiopathic subgroup we can see similar results, with significantly higher rates of ST elevation (31% in the first event vs. 11% in the recurrence; *p* = 0.003), Knuckle sign (40% in the first event vs. 14% in the recurrence; *p* < 0.001), and ST elevation larger in lead L2 than L3 (25% in the first event vs. 2% in the recurrence; *p* < 0.001) (Table 2).

There was a significant difference in the presence of a pericardial effusion deemed by an echocardiography specialist to be any size beyond small, either by visual estimation or measurement, between the first event and the recurrence (30% in the first event vs. 11% in the recurrence; *p* = 0.02). For the idiopathic subgroup, we were able to see a significant difference as well (32% in the first event vs. 8% in the recurrence; *p* = 0.006).

The peak CRP levels during the first event were greater than those documented during the recurrence (mean 97 mg/l vs. 66 mg/l, *p* < 0.001). Only one patient had a normal CRP level (< 5 mg/l) during the first presentation. Three other patients had a normal CRP level during the recurrent event. Similar findings were seen in the subgroup of patients with idiopathic pericarditis (mean CRP 68 mg/l in the first event vs. 45 mg/l in the recurrence; *p* = 0.003).

**Fig. 2:**
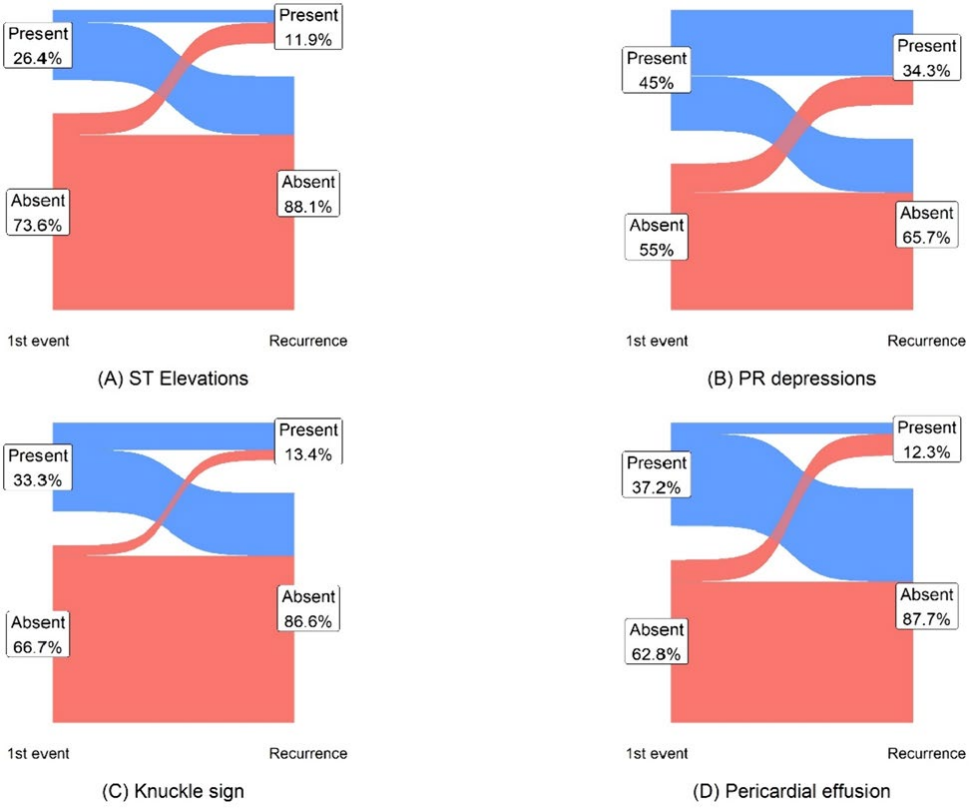
Prevalence of electrocardiographic signs and a significant pericardial effusion in the recurrent event, by presence in the initial presentation

There was a certain amount of cross-over between the groups of patients who had certain ECG findings or a significant pericardial effusion, though these signs were mostly more common in those who demonstrated these signs in the first event (Fig. 2). In patients who were negative for ECG signs and had a significant pericardial effusion, up to 20% had these findings present during AP recurrence.

## Discussion

In this study, we compared the clinical and laboratory characteristics between the first presentation of AP and the first documented recurrence. Our main finding is that ECG signs and pericardial effusion are less present or attenuated during recurrent events when compared to the first presentation. Also, serum markers of inflammation are increased to a lesser extent during recurrence. The presence of these findings was mostly not associated with initial disease management. In addition, we describe up to 20% of cases that had positive findings during their recurrence were negative for these findings during their index event. We suggest that the discrepancies in these cases are mainly caused by the timing of evaluation, but this is more speculative and the nature of this phenomenon, as well as its clinical and prognostic significance, warrant further evaluation. On the other hand, the majority of cases do show less pronounced findings that cannot be explained by timing alone, such as maximal CRP levels.

The diagnosis of AP recurrence is a challenge for clinicians. The significance of correct diagnosis is earlier initiation of management, and more rapid symptom relief. Our findings suggest that the sensitivity of the clinical parameters included in the diagnostic criteria supported by current guidelines for RP is low. It seems that in practice, much more emphasis is given to the recurrence of symptoms and the use of serum markers of inflammation. Many patients are diagnosed without meeting the rigid formal guideline-based criteria, most commonly based either on the recurrence of typical pain or pericardial effusion, accompanied by elevated inflammatory markers. They are treated with anti-inflammatory medications and mostly respond well to therapy. Though many contemporary cases go on to get ambulatory cardiac CMR studies that are much more available, we were unable to collect advanced imaging data in this historical cohort. In our experience as a tertiary referral center with a dedicated outpatient clinic for pericardial disease, this approach is useful when deciding on patient management, although some patients do present with persistent chest pain with no other objective evidence of active inflammation.

The low rates of significant pericardial effusion for example, suggest that evaluation with means beyond a bedside echo in the clinic or ED is not necessary in most cases. This understanding can potentially increase confidence in managing such cases in the outpatient setting, thereby reducing the burden of unnecessary hospitalizations and the associated costs of outpatient echo studies. This seems to be a valid strategy at least for patients with a RP, who are otherwise stable and are not at risk of rapid effusion accumulation (e.g., anticoagulant therapy) (Table 2).

**Table 2:** Comparison of the clinical parameters during acute pericarditis and the recurrent event: idiopathic subgroup

	First event	Recurrence	*p* value
Mean initial CRP levels (mg/l)	47 ± 54	32 ± 38	0.05
Mean maximal CRP levels (mg/l)	68 ± 77	45 ± 54	0.003
ST elevations *n* (%)	28 (31%)	10 (11%)	0.003
PR depressions *n* (%)	42 (47%)	31 (34%)	0.07
Spodick’s sign *n* (%)	20 (22%)	13 (14%)	0.21
Knuckle sign *n* (%)	36 (40%)	13 (14%)	< 0.001
ST elevation L2 > L3 *n* (%)	22 (25%)	2 (2%)	< 0.001
Pericardial effusion > small *n* (%)	26 (32%)	3 (8%)	0.006

CRP, C-reactive protein

Furthermore, our findings, that the manifestation of RP tend to be more subtle, should encourage clinicians to be more proactive in pursuing the diagnosis when it’s unclear, and the patient presents with persistent pain despite a lack of further objective evidence of active inflammation. In these instances, the use of advanced imaging techniques such as CMR can aid in decision making [10]. This could be critical, especially since ongoing pain may lead to up-titration of anti-inflammatory, corticosteroid, or immunomodulatory therapy.

This study has several limitations. First, this single-center retrospective observational study used ICD9 diagnosis coding to identify potential patients. Due to software issues, we were unable to detect PPS patients who did not have symptoms or recurrence beyond the initial hospitalization in the cardiothoracic surgery department. Data on recurrent events treated in other facilities were not available for inclusion in our analysis. Although all cases were reviewed manually to assure that the adjudicated diagnosis was adequate, we did allow for some amount flexibility with the definition in recurrent cases, which could also be defined according to concurrence of symptoms along with deranged laboratory biomarkers, as well as advanced imaging findings. We do believe that this was a more realistic approach to diagnosis, and we also believe that future guidelines will be in line with some form of similar definition, as expert opinion and novel clinical trials in the field have done so as well. We were also unable to obtain exact documentation of time from symptom onset to presentation, or proper documentation of the physical examination detailing the presence or absence of a pericardial friction rub, in either event.

## Conclusions

The clinical manifestations seen in recurrent events of AP seem to be attenuated compared to the first acute presentation. Future studies should focus on the usefulness of serum inflammatory markers and advanced imaging in establishing the diagnosis, as well as in identifying individuals who would be better served with outpatient management.

## References

[1] Chiabrando JG, Bonaventura A, Vecchié A (2020). Management of acute and recurrent pericarditis: JACC state-of-the-art review. J Am Coll Cardiol.

[2] Adler Y, Charron P, Imazio M (2015). 2015 ESC Guidelines for the diagnosis and management of pericardial diseases. Eur Heart J.

[3] Chaubey VK, Chhabra L (2014). Spodick’s sign: a helpful electrocardiographic clue to the diagnosis of acute pericarditis. Perm J.

[4] Lopera-Mejía L, Ocampo-Moreno D, Lopera-Cardona S, Ospina-Soto S, Duque-Ramírez M (2022). aVR: the forgotten lead. Rev Colomb Cardiol.

[5] Henning D, Moeller CM, Fjaeldstad A, Fogel M, Fischer C, Ullman E (2012). Evaluating the utility of ST elevation in lead II > lead III in differentiating pericardial disease from STEMI. Scand J Trauma Resusc Emerg Med.

[6] Imazio M, Andreis A, Lubian M (2021). The Torino Pericarditis Score: a new-risk stratification tool to predict complicated pericarditis. Intern Emerg Med.

[7] Dh S (1917). Differential diagnosis of acute pericarditis. Prog Cardiovasc Dis.

[8] Bizzi E, Picchi C, Mastrangelo G, Imazio M, Brucato A (2022). Recent advances in pericarditis. Eur J Intern Med.

[9] Imazio M, Lazaros G, Gattorno M (2022). Anti-interleukin-1 agents for pericarditis: a primer for cardiologists. Eur Heart J.

[10] Andreis A, Imazio M, Casula M, Avondo S, Brucato A (2021). Recurrent pericarditis: an update on diagnosis and management. Intern Emerg Med.

[11] Bonaventura A (2022). The long journey of interleukin-1 in acute and recurrent pericarditis. Eur Heart J.

[12] Lazaros G, Antonopoulos A, Lazarou E, Vlachopoulos C, Tsioufis K (2021). The tale of refractory recurrent pericarditis. Intern Emerg Med.

[13] Massaro MG, Gallo A, Montalto M, Manna R (2023). Treatment of recurrent pericarditis in elderly. Eur J Intern Med.

[14] Chetrit M, Xu B, Verma BR, Klein AL (2019). Multimodality imaging for the assessment of pericardial diseases. Curr Cardiol Rep.

[15] Chetrit M, Xu B, Kwon DH (2020). Imaging-guided therapies for pericardial diseases. JACC Cardiovasc Imaging.

[16] Young PM, Glockner JF, Williamson EE (2012). MR imaging findings in 76 consecutive surgically proven cases of pericardial disease with CT and pathologic correlation. Int J Cardiovasc Imaging.

[17] Imazio M, Demichelis B, Parrini I (2004). Recurrent pain without objective evidence of disease in patients with previous idiopathic or viral acute pericarditis. Am J Cardiol.

